# Incarceration and Family Stress as Understood through the Family Process Theory: Evidence from Hong Kong

**DOI:** 10.3389/fpsyg.2016.00881

**Published:** 2016-06-14

**Authors:** Wing Hong Chui

**Affiliations:** ^1^Department of Applied Social Sciences, City University of Hong KongHong Kong, China; ^2^Institute of Advanced Study, Durham UniversityDurham, UK

**Keywords:** family process theory, incarcerated fathers, transitions, rituals, ambiguity, fatherhood

## Abstract

The myriad of negative effects brought about by the incarceration of a family member have consistently been demonstrated in research. However, previous works have tended to focus on the perspectives of family members separately, rather than exploring the dynamic relationships within the family as an entire unit. Moreover, such research is still limited in the Chinese cultural context. Thus, the current study aimed to examine the applicability of the Family Process Theory on a small sample of Chinese fathers who were imprisoned and have returned to their communities, conceptualizing the challenges faced by their families under the theoretical elements of rules of transformation, rituals, ambiguous loss, and boundary ambiguity. A total of 17 participants were recruited through the assistance of service organizations, and data were collected via semi-structured interviews. As congruent with the theory, inmates’ families were found to come across problems particularly in the transitioning phase and establishment of new rituals, while ambiguity surrounding the incarceration and the blurred role of the father led to problems with fatherhood identity both during and after the incarceration. Recommendations are made, in line with these findings and the suggestions of ex-prisoners, for services to have more of a focus on preserving the integrity of inmates’ families. Given the right circumstances, families can even serve as a motivator for incarcerated fathers to improve themselves and mature, leveraging the important supportive role of the family and the fear of again being separated from one’s family as effective deterrents to recidivism.

## Introduction

The impact of incarceration on families has received increased attention from researchers in recent years (King, 1993; Myers et al., 1999; Hairston, 2001; Kazura, 2001; Magaletta and Herbst, 2001; Schen, 2005; Codd, 2008; Murray et al., 2009, 2014; Shlafer and Poehlmann, 2010; Graham and Harris, 2013; Manby et al., 2015). The increasing size of the prison population, together with findings revealing both acute and prolonged impacts on life trajectories of family members with at least one imprisoned parent in the family, were major factors contributing to growing interest in the field (Goffman, 1961; Schwartz and Weintraub, 1974; Brodsky, 1975; Swan, 1981; Jorgensen et al., 1986; Hairston and Lockett, 1987; Hairston, 1998, 2001, 2004; Casey et al., 2015). For instance, it has been found that incarcerated parents were more likely to have children who developed behavioral delinquency, came across academic hardship, and suffered from psychological distresses (Bloom, 1995; Johnston, 1995; Kampfner, 1995; Boswell and Wedge, 2002; Bendheim-Thoman Center for Research on Child Wellbeing, 2008; Murray and Farrington, 2008; Wildeman, 2009; Chui, 2010; Harris et al., 2010; Shlafer and Poehlmann, 2010). Moreover, couples with an incarcerated member unsurprisingly had lower rates of continuation with the relationship and marriage (Western et al., 2004; Western, 2006) and were also further associated with heightened likelihoods of divorce, increased levels of domestic violence, greater economic strain, and a higher chance of partners engaging in extramarital sex (Siennick et al., 2014).

The amount of evidence describing the myriad negative consequences of incarceration on family members has been abundant. However, previous studies have tended to approach the topic from singular perspectives, such as focusing on the incarcerated individual or certain family members separately rather than investigating the dynamic interactions between them. For the purposes of developing more effective policies and strategies to help all family members affected by incarceration, it is essential to have a comprehensive understanding of the challenges faced by such families, which the current study aims to contribute to. The scope of the current study is focused on incarcerated fathers, this being reflective of the current reality wherein males constitute a much higher proportion of the prison population, and also due to the key role fathers play to members of a family, which is especially the case in societies that are still more patriarchal (see also Chui, 2016).

In this paper, the family process theory is chosen as the framework for analysis because it specifically relates to cultural values in a Chinese context. Despite the fact that Hong Kong has been subject to Western or British influences, a number of scholars argue that the traditional family values and beliefs still hold a central position in the Chinese culture. For instance, the norms of familial obligation, family support, family harmony, interconnectedness, and parental strictness and kindness have all been endorsed by the Chinese across the world (see, for example, Wang et al., 2007; Li and Lamb, 2013). In a patriarchal society like Hong Kong, the man in the family is often regarded as the breadwinner who provides for and maintains the family, and the man, especially the father, is given all power in making decisions for his wife and children about important matters on various daily life situations. However, these norms and roles of the father may change as a result of his criminal behavior and incarceration. Not only does his criminality and imprisonment bring shame to the family but it also, in turn, affects the dynamics, communications, rituals, and processes within a family. In this respect, the family process theory would help shed light on how each member of the family influences each other. It, more specifically, argues that family members are interconnected and interdependent individuals, thereby explaining the changing roles of each individual as a result of changing life events and family circumstances.

### The Family Process Theory

Apart from conceiving the functioning of family members as the outcome of individual efforts, it can be understood as both the product of and actor on family processes. In other words, family outcomes are not merely guided by individual contributions, but rather the dynamic interactions between members. It can be observed that some families fare better than others in the face of one of its members being incarcerated, and Family Process Theory can be applied to explain why this may be the case.

Family Process Theory (also known as Family Systems Theory) is a theory that conceptualizes the family as a complex, interacting, homeostatic system that strives to maintain equilibrium (Broderick, 1993). Several notions are proposed, namely that: all elements of the system are interconnected, understanding it is only possible when viewing it as a whole, the system interacts with the environment via a continual feedback loop, and that the system is a heuristic of understanding rather than an actual tangible structure (White et al., 2014). Simply put, the theory asserts that the focus of analysis should be on the interactions among family members instead of each individual (Day et al., 2006), addressing the emergent properties of these relationships (Broderick, 1993). Family Process Theory is better understood as a broad umbrella approach treating families as systems connected by interacting processes, rather than a precise explanatory theory with specific fixed dimensions. As such, various researchers throughout the years have adopted approaches congruent with the theory, even without making explicit mention of it (e.g., Carlson and Cervera, 1992). Through a comprehensive review of the literature, Dyer et al. (2012) identified four elements of the theory particularly relevant to the context of incarcerated fathers, namely rules of transformation, family rituals, ambiguous loss and boundary ambiguity.

Firstly, rules of transformation are defined as ‘protocols’ held by family members determining how they interact with each other and the environment (White et al., 2014). Whether or not families share similar rules of transformation when dealing with environmental inputs has been debated (Boss, 2006), though it is nonetheless acknowledged that they exist and are crucial in predicting family outcomes. For instance, the withdrawal of the father from the system via incarceration represents an input to the family, with family outcomes then being dependent on whether the family possesses either pre-existing rules to deal with this change or the flexibility to cope with this transformation and re-attain a state of equilibrium (Broderick, 1993; Burr and Klein, 1994). However, as incarceration is not a socially common occurrence, it is unlikely for families to have rules in place to respond to its occurrence (Clarke et al., 2005); that families struggle to cope following such an event is hardly surprising (e.g., Fishman, 1990; Arditti et al., 2003a,b). For example, if the father is removed from the process, the family is likely to experience increased distress due to financial instability and weakened connection among family members (Fishman, 1990; Arditti et al., 2003a,b).

The second element of focus is family rituals, which can be understood simply as the routine practices and interactions that family members engage in. Family rituals are proposed to contribute to good family functioning, strengthening the collective identity of a family by increasing intimacy among members (Bossard and Boll, 1950; Doherty, 1997; Day, 2002; Fiese et al., 2002). However, rituals are severely hampered in the event of an incarcerated person due to the imposition of prison regulations regarding the time and context under which inmates are allowed to interact with their families (Schneller, 1976). In the end, some families may give up by terminating visits, not reading letters, and stopping telephone calls from the incarcerated person (Hairston, 1998). The inability to practice rituals may lead to the failure of a family to maintain equilibrium, consequently weakening it.

Ambiguous loss and boundary ambiguity make up the other two important aspects of family processes highlighted here. Within the current context, ambiguous loss relates to situations wherein the circumstances surrounding the physical or psychological loss of a family member have not been clearly communicated (Boss, 2002). It can be experienced at and across different stages of the incarceration process: beginning with the arrest of the father, carrying over to the procession of legal arrangements, continuing throughout the duration of the father’s imprisonment, and even persisting upon his release (McDermott and King, 1992). On the other hand, boundary ambiguity refers to the uncertainty of family members regarding the roles they are supposed to play in the family. The withdrawal of the father from the family is not merely a physical one, but families may struggle on how to incorporate them into the new rituals of the family (e.g., Schwartz and Weintraub, 1974; Jorgensen et al., 1986; Boswell and Wedge, 2002; Bates et al., 2003; Clarke et al., 2005). The uncertainty may grow to a point where children no longer recognize the father as part of the family (Bates et al., 2003), though this may actually be perceived as a means to alleviate stress as boundary ambiguity will be reduced upon clearly defining that the father no longer plays a fatherhood role (Boss, 2002). Research on incarceration thus far has indeed uncovered the prevalence of both types of ambiguity in families of the incarcerated (Arditti et al., 2003a,b; Arditti, 2005; Roy and Dyson, 2005), though the distinction between ambiguous loss and boundary ambiguity has not always been operationally made (Boss, 2007; Carroll et al., 2007). Nonetheless, it is important to separately define these two concepts as they may not always be related congruently. For instance, if the father had not taken on a fatherhood role prior to his withdrawal from the family via incarceration, boundary ambiguity would remain low even in the case of ambiguous loss. In contrast, although families reduce ambiguous loss through clear communication of incarceration circumstances, they may still struggle with high boundary ambiguity in the absence of appropriate strategies on how to retain familial roles (Dyer et al., 2012).

The Family Process Theory provides researchers with a general theoretical framework under which the challenges faced by families with an incarcerated father can be better conceptualized, and ideally addressed in practice consequently. The current study aims to garner support for the applicability of the theory to the population of families of incarcerated fathers, by interviewing a sample of Chinese ex-inmates. This will have the additional benefit of bolstering knowledge of the region, as research on the families of the incarcerated in Chinese contexts remains limited; it is also hoped that insights will be drawn from studying a sample rooted in a collectivist culture, as this may have some bearing on the dynamics of family relationships observed.

## Materials and Methods

A qualitative approach was employed to facilitate a conversational space for individual ex-inmates to talk about their unique experiences of fatherhood and felt family stress during and after imprisonment. Based on the author’s experience with this unique group of participants (Chui, 1999, 2003, 2016; Adorjan and Chui, 2012, 2014; Chui and Cheng, 2013, 2014), a semi-structured interview protocol was followed, involving a set of open-ended questions and guiding questions, to tease out and explore further the narratives with regard to the felt family stress, coping strategies, and the negotiations involved in preserving or transforming their fatherhood identity. Given the sensitivity and complexity of the issues discussed, I took appropriate measures to facilitate an open conversational space where they felt safe and comfortable to disclose their innermost thoughts, feelings and reflections. This was accomplished by devoting 20 min or more for rapport building with the participants (Creswell and Miller, 2000). For example, the participants were asked to introduce themselves and their interests in volunteering to participate in this research. Throughout, the course of rapport building and the interview, I kept an open mind to listen to their life experiences and reflections. Without being judgmental, clarification, elaboration, and probing were sought whenever I encountered some form of value conflicts, contradictions, uncertainty, or ambiguity in their responses. By no means were these probing judgmental or reflections of my own assumptions, beliefs and biases, rather I probed further to learn more about their lived experiences, which are novel to me, and to avoid any room for misinterpreting their narratives.

### Participants

The target population of the current study was incarcerated fathers but they have returned to their communities when the interview was conducted. Interested individuals were referred by social workers from different non-governmental organizations and extended an invitation to participate. The final sample comprised a total of 17 ex-inmates ranging from 33 to 65 years-old. With regards to the crimes they had been incarcerated for, the majority of them had done time for drug-related crimes (*N* = 10), two for robbery, and one each for the crimes of indecent assault, blackmail, theft, attempted manslaughter, and breach of probation order. Their length of incarceration ranged from 1 to 22 years, though the latter was an outlier; upon removing the 22-years sentence from consideration, the mean incarceration length of the rest of the participants was 3.4 years.

### Procedures

As part of the referral process, social workers obtained signed consent from participants indicating their interest and willingness to participate in the study. Following this, arrangements were made for participants to attend an individual interview, which was conducted in an enclosed counseling room free from external distractions. Prior to the commencement of each interview, the interviewer informed the participant of the purposes of the study, and once again obtained both verbal and written consent from them to ensure their willingness to participate as well as obtain permission to record the interviews. Participants were also assured that they were free to withdraw at any time without any penalty, and that their results would be kept strictly confidential. Ethical approval for data collection was obtained from the Human Research Ethics Committee for Non-clinical Faculties of The University of Hong Kong where the author was previously employed.

All interviews were conducted either by the author or a research assistant who had been trained by him. Cantonese was used as the medium of communication as it is the primary dialect of Hong Kong. Interviews were semi-structured and were designed to cover the following broad categories: (a) the types of services that ex-inmates had received and their relationship with said services; (b) details of their lives during their period of imprisonment; (c) the impacts of their incarceration on the lives of their partners/spouses; (d) the impacts of their incarceration on the lives of their children; (e) the strategies employed by them to prevent their re-engagement with crime; and (f) recommendations on how the assistance and services provided to inmates can be improved. Examples of questions include: ‘How did you comment on the impact of your incarceration on your family members and your parenting as father?,’ ‘How did you see your role as a father before, during and after incarceration?,’ ‘How did you feel when your partner and child(ren) visited you in prison and after your return to your family or community?,’ and ‘What are your views on how policies or programs should be delivered to promote positive relationships among inmates or inmates and their children?’ All participants completed the interview in its entirety. Interviews lasted between 60 and 120 min, the average being 90 min.

### Data Analysis

Recorded interviews were transcribed verbatim, and then translated into English. Verbal reports recorded were transcribed as verbatim. Thematic qualitative analysis was used to analyze the data, in accordance with the widely used procedural guidelines laid out by Braun and Clarke (2006). The six phases of the thematic analysis suggested by Braun and Clarke were followed very closely when analyzing the qualitative data. These six phases are: familiarizing myself with the interview data, generating initial codes, searching for themes, reviewing themes, defining and naming themes, and producing the report. ‘Repeated reading’ of the transcriptions enabled me to be familiar with the depth and breadth of the qualitative data. While all transcriptions were read for at least three times, there were several occasions I needed to check them back against the audio-recorded file for accuracy, and began to take notes and generate ideas whenever appropriate (Braun and Clarke, 2006, p. 88). The family process theory, in many ways, helped guide the thematic analysis of interviews to generate codes and search for themes. Initial codes, such as ‘incarceration,’ ‘challenge,’ ‘stress,’ ‘rules,’ ‘transformations,’ ‘rituals,’ ‘routines,’ ‘interactions,’ ‘functioning,’ ‘loss,’ ‘boundary ambiguity,’ and ‘re-establishment of the identity’ were generated from the said theory. In this regard, the process of coding was ‘theory-driven’ rather than ‘data-driven.’ Several highlighters and colored pens were used to code manually in order to identify codes and match them with data extracts. To ensure that all data had been given full attention, two trained research assistants first coded and collated data individually, and then I worked with them to examine those discrepancies and inconsistencies across their codes. The next phase was to organize those codes to form main themes and some to sub-themes, and all that will be presented in the next section. Several mind-maps were drawn to tease out the complex or inter-relationships of these themes and sub-themes, as well as account for any inconsistencies in the data, by adding or dropping some codes after several rounds of discussion. The process of reviewing and defining themes and sub-themes was indeed very time-consuming and labor intensive, and yet was necessary to ensure all data being given equal attention and the stories shared by the participant being presented accurately. Data extracts below are reproduced verbatim with occasional grammatical changes that correct for the translation without sacrificing content.

## Results

### Rules of Transformation

First and foremost, it was unsurprisingly evident that incarceration posed a huge challenge to inmates and their families, that a majority of them reported having problems adjusting to. Participants commonly described the adverse impacts of their sudden withdrawal on their family members:

“There’s no man in home to look after all kind of things, there’s no man to make decisions, there’s no father and no husband staying with them.”

“[My wife] was frightened and helpless. My imprisonment was so sudden to her. She couldn’t predict it.”

“[My children] lost a father, the environment was changed suddenly, they lost the sense of purpose. Before I went to jail, their father could make decisions for them. After I went to jail, they needed to make decisions on their own.”

“[My wife] felt nervous and frustrated before I was sent to jail as she didn’t know how long the sentence would be; when I was in jail, she had to solve every problem by herself. Besides, she had to visit me and waited when I left prison, she didn’t know what I was going to do. I think she was helpless.”

Naturally, the struggles faced by families transitioning into this new phase brought about various problems, most notably relating to finances and stigmatization. In worst cases, the failure to transition led to the complete collapse of the family:

“[My wife] loves me. She relied on me. When I was in prison, I could feel that she felt helpless. Such as [that she couldn’t afford] the living expenses.”

“My wife borrowed money from others, relatives and friends. But people won’t lend money to you anymore as you borrow too many times.”

“My wife couldn’t read Chinese. She didn’t know how to fill the forms (for social subsidy).”

“She just arrived at Hong Kong. She didn’t have any relatives here. She doesn’t speak Cantonese. It was difficult for her to find jobs.”

“She didn’t need my money. [The largest impact was to] her dignity and her mind: when others knew that her husband was in prison, the damage was tremendous.”

“For example, she had to get my signature, otherwise our son couldn’t take part in the competition. His future would have some negative impacts. If I was not in jail, then I could sign it by myself. …… My son cares about his face very much. It must have had some negative impacts on his soul.”

“[My wife] left after I had been in prison for 2 or 3 months.”

“[The incarceration] has made me lose everything. For example, my family has been broken. I got divorced, and the relationship between me and my children has become poorer.”

It is understandable that affected families may not have possessed appropriate rules of transformation to adapt to this significant life event; given the unique nature of incarceration. Nonetheless, of the families that eventually managed to adapt to it with some degree of success, it could be observed that external support played a key role in their adaptation, be it from relatives, social workers or organizations:

“My wife was from mainland China. She couldn’t get a job. Therefore, she worked at my brother’s company. They paid her to work there.”

“The family has expenses. My mother helped to support my family by getting a part-time job.”

“Miss Wong (a social worker) had guided her to many places while teaching her how to live in Hong Kong. When she visited me, I would teach her as well.”

“My brother helped [my wife] visit the social worker. The social worker helped us a lot. She helped my wife to get a job.”

“[My wife] got Comprehensive Social Security Assistance and found social workers to share her problems with. She contacted [the name of the non-governmental organization]. It was useful. Social workers there talked with my wife and visited me together with my wife.”

Thus, this implicates social support as a factor bearing an important influence on the adaptability of inmates’ families following their incarceration, and is an element that should be bolstered.

### Rituals

When it came to rituals, it could again be seen that incarceration disrupted families’ ability to engage in habitual activities, which is to be expected:

“I didn’t have the ability to take him shopping. We couldn’t play activities like playing badminton.”

“He used to see his father every day. Then his father was put in jail suddenly. It harmed his mind. … He once wanted to leave the family.”

“There are feelings after my family has broken. Especially for the Chinese, feelings become stronger during festivals.”

Interestingly, many participants made little to no mention of the disruption to their family rituals brought about by their incarceration. This could be reflective of the fact that participants may not have had the best relationships with their family members to begin with, perhaps due to their engagement in criminal activity that landed them in jail in the first place.

Nonetheless, many families attempted to establish new family rituals in a bid to retain the bonds between them. They did this via methods such as visiting and writing regularly:

“[My brother and his wife] visited once every 2 months. I could have someone to talk with when I was depressed and when I was suffering. They comforted me to not be sad when my wife left me. They told me that there were chances everywhere. I still had a chance. My brother liked to say that.”

“[I wrote to her] twice a month. It’s a kind of sympathetic letter. I would like to comfort her through letters. I knew my wife was busy and she seldom wrote letters. She seldom picked up a pen, but she would prefer visit me in person. [She visited] twice a month.”

“I sent out 1–2 letters every month. I wrote to my wife and son, but I put my son’s name on the envelope. I wanted to communicate with them.”

“Yes, this [communication method] could help. I must be a father and husband.”

These rituals helped participants and their families maintain communication and work through their problems to some extent, which also facilitated inmates’ taking on of a fatherhood role. Moreover, the mere feeling of supportiveness garnered by these simple rituals could be inferred from participants who initially expressed reluctance at the idea, or who did not necessarily see any practical value in it, who cherished their families’ visits regardless:

“I told her that she could choose not to visit me. She visited me once per month but I told her not to. (*Interviewer: Did you actually want her to visit you?)* Of course, I would be happier when I saw her.”

“I wanted to see her. …I wanted to see them, although I didn’t know what to say.”

However, it was not easy to establish such rituals due to reasons of practicality or limitations in prison visitation policy:

“I told her if there’s no problem, it would be the same to write letters. I thought it was too hard to bring along the child and to walk a long distance to get to the prison. They had to walk a long distance after getting off the bus.”

“My ex-wife would bring my son to visit me. The visitation time was limited. (*Interviewer: How long was the visitation time?)* 15 min.”

“[My wife visited] once per week. She got on the earliest ferry at 5 am, and visited me for 15 min. When she got home [at the end of the day], it was at night. She was tired. The distance was too long. I felt guilty.”

### Ambiguous Loss and Boundary Ambiguity

Ambiguous loss was a common theme for the families of the incarcerated. The majority of participants either withheld incarceration-related information from their children, or simply chose not to approach the topic themselves:

“I left for a very long time this time. [My daughter] always couldn’t see me. Her mother told lies to her that I was working in other places.”

“I told [my son] that my life was fine in prison. I wouldn’t tell him the truth that my life was sad and that I received tranquilizers. I won’t tell him those truths. He didn’t understand.”

“No, [my son] doesn’t know. I didn’t want him to know. There is no impact to him if he doesn’t know about this. He will have impacts when he knows it. If the father is such a bad person, he will give up more easily.”

“(Talking about the personalities of his daughters after his incarceration) Unlike in the past, they were passive, quiet and shy. They didn’t see me for 13 months. I think they might have known that I was in prison. (*Interviewer: Did you explain your situation to them?*) No. They didn’t ask as well.”

This seemed to be somewhat linked to boundary ambiguity, in that the disconnect brought about via ambiguousness of the situation served as an invisible barrier between father and children. In general, participants commonly described the distancing of themselves from family members:

“In the beginning, nobody came to visit me in prison. I have a wife, and I also have a daughter and a son. I don’t know whether the two children knew [about my imprisonment]. I don’t know whether my wife told them.”

“I didn’t know how they were carrying on their life, and they would be very helpless.”

“[My wife] was helpless indeed. She couldn’t handle the problems of my daughters, as my daughters only conform to what I say. Therefore, I don’t know how my wife handled the problems.”

“(*Interviewer: What do you think you could have done during your imprisonment in order to keep in touch with your family?*) During imprisonment? No, nothing. (*Interviewer: Do you think you took the role of a father*) No one taught me how in the prison. It is not my first time being imprisoned.”

This led to difficulties in re-establishing participants’ fatherhood identity upon their discharge, due to the ambiguity of their role in the familial unit:

“When I was first discharged, both of them, my son and daughter were not willing to talk to me. (*Interviewer: did your children know why you went to prison?*) I don’t know. I don’t know whether they knew it. I don’t even know whether my wife told them. I didn’t tell them. (*Interviewer: Do you think the incarceration had any impacts on your children?*) I don’t know if there was any impact. I haven’t asked them and my wife hasn’t told me about that.”

“For the elder [son], the relationship is not so good. He can’t do what I expect, although I was not restrictive to him. I fought him seriously. The main cause is that he didn’t listen to me at all.”

“I can’t teach [my son]. I can’t treat him as my friend. I need to be a father when facing him. I have tried to treat him as my friend, but I found that he won’t listen to me. He’s not willing to obey me. Now, the son hates me. … Whatever he wants to do, I will let him do.”

However, in exceptional cases, participants were very forthcoming and open with their children about the circumstances of their incarceration, minimizing ambiguity. In such cases, these participants managed to retain their sense of fatherhood whilst also making use of their experiences to teach their children:

“I let them know all. This was the consequence of my offending the law. I would like to let them know the truth.”

“I even told him everything from my entrance to the society to his birth until now… I have told him all these things about the lessons of life. I have to be frank.”

“My children knew what had happened when they were growing up. For example, my younger child asked me why I was staying in prison when he visited me. I said because I was naughty, and I had been caught to wash clothes here. Therefore, I asked him to be a good boy. He understood. I think if I lie to him, he will change his view on me. I never lie to him.”

### Insights for Future Directions

In fact, upon being asked what steps could further be taken to assist inmates moving forward, many participants made suggestions revolving around the preservation and enhancement of family relationships, highlighting its importance:

“I think that the government can financially support more social work services for ex-prisoners. These services do help to enhance the family relationship.”

“I hope that there will be more programs to [teach me] how to become a good father during and after imprisonment.”

“The most important point is the government provides this kind of help. Approach their families. Contact their families or ask the social workers to contact those families.”

“Prisoners should get closer to their family. It’s important for prisoners to be able to meet their children, just using the phone is useless. They should allow family members to bring food to prisoners. They should organize more activities.”

“They can organize family days held in prison. … When the offenders performed well, they were offered a family day per month. However, this practice wasn’t found in other prisons. The family day does help to maintain the family relationship.”

Indeed, although imprisonment was a great hardship that took its toll on inmates and their families, a number of them managed to take something positive away from the experience, in that prison had taught them to be appreciative of their family, and that the fear of being separated from them again served as an effective deterrent preventing them from engaging in criminal behaviors again:

“Although I know selling drugs can earn a lot of money, I was scared by the length of the sentence. I was not scared at all before my wife arrived in Hong Kong. Now I am, because I am concerned about my family. I don’t know what to do if I were sent to jail again.”

“I know myself. I realize the consequence of separating with [my family].”

“I have thought about the feelings of my family, that these are the effects that I brought to them due to my imprisonment.… I have to care about feelings of my family members.”

“In the past, I lived in Hong Kong, lonely, without anyone’s care; now, I am with my family, and it’s different.”

Moreover, as explicitly identified by one participant, the provision of love played a key role in his turning over a new leaf:

“When I was first released, I couldn’t contain my feelings of wanting to take drugs. But I couldn’t offend the law, so I was forced to seek help from Ms. Yeung (a social worker). But Ms. Yeung really touched me. She cared very much. Her love touched me. Therefore, I don’t want to fall down again because I don’t want to discourage her.

Because many drug addicts have lost love already, more love and care can be given to them. If they accept help, they can be helped.”

Given the right conditions, families could constitute an important source of love and support, elements that are integral for helping offenders get back on their feet. In this regard, family could even serve as a motivator for inmates to improve themselves. These notions are summed up nicely in the following statement from one insightful participant:

“Prisons are [beneficial] for those who have strong determination, whose families are not problematic; prisons may be useful since [prisoners] don’t want to lose anymore. But for some people, it may not be the same case; especially to those who don’t have a family, who lost care and love. They give up on themselves.”

## Discussion

To reiterate, the central tenet of family process theory is that the family should be viewed not merely as the sum of its individuals, but rather as a collective unit that undergoes both internal and external interactions – between each other and the environment – in a bid to achieve and maintain equilibrium. By applying this theory to the population under enquiry, this afforded a better understanding of some of the mechanisms by which incarceration poses challenges to those families affected by it, which allows for some recommendations to be made to improve the whole process for them.

Firstly, it was observed that families generally lacked the appropriate rules of transformation to deal with the incarceration of the father. This was to be expected as, given the unique and uncommon nature of incarceration, knowledge and guidance on moving forward from it is still limited (Arditti et al., 2003a,b; Clarke et al., 2005). Congruent with previous research, the jolting withdrawal of the father led to key problems namely pertaining to perceived stigmatization (Fishman, 1988; Phillips and Gates, 2011) and finances (Arditti et al., 2003a; Schwartz-Soicher et al., 2011). These two issues are likely particularly pertinent within the Chinese cultural context: firstly, Chinese traditionally hold negative views toward anything associated with prison; secondly, Chinese males typically remain the sole breadwinners of the family, which may cause their wives to be ‘over-reliant’ on them with regards to certain things (Chui, 2010), as was observed in this study. Moreover, a commonly reported sentiment by the research participants was that their incarceration was very sudden to their families both practically and emotionally, implying a lack of preparedness on their part. Taken together, these findings suggest that the period of transition following the incarceration was particularly jarring for families of inmates, and that more should be done to help them adequately adapt during said phase, especially since it was during this sensitive stage that families seemed most vulnerable to collapse. This recommendation is bolstered by the further finding that, of the families who somewhat successfully navigated this tenuous period, nearly all of them did so with the assistance of external support, sources being relatives, social workers or non-profit organizations. As such, this strengthens the notion that social support could have an integral influence on families’ adaptive rules of transformation in the face of incarceration.

Moving on to the aspect of rituals, although not all participants went into much detail on their rituals prior to their incarceration, there was nonetheless some evidence that incarceration disrupted, if not completely broke, the routines of families. For instance, on several occasions, the research participants, who reported having close family relationships, mentioned that routines of families like shopping, playing badminton, having yum-cha, and celebrating festive reasons were stopped since their imprisonment. This finding is to be expected given the fact that incarceration can disrupt stable family formation and functioning, and is in line with those of previous works (Schneller, 1976; Doherty, 1997; Day, 2002; Fiese et al., 2002). As stated by one participant, the feelings of loss were particularly strong during festivals such as the Chinese New Year, which is widely recognized as symbolizing the cherished Chinese values of connectedness and unity. In this respect, family rituals may be particularly relevant and important to Chinese contexts, as Chinese culture is traditionally collectivist and places much emphasis on kinship and family bonds, which family rituals play a key role in maintaining. In fact, this may also partly explain why many inmates’ families made the effort to establish new rituals upon their incarceration, despite them not necessarily being on the best terms beforehand. As echoed in the literature (e.g., La Vigne et al., 2005), this was met with great benefit: said inmates consistently described the positive effects of such rituals in helping them work through their problems or retaining their role in their family, or simply being a source of support. Thus, while trying to improve avenues of support for inmates and their families, it may prove valuable to firstly recognize the importance of family rituals, and to further facilitate the establishment of new rituals following the incarceration. The latter may be done by adjusting mail-writing and visitation policies to be more flexible and accommodating, particularly since participants also mentioned the difficulties involved in their family members’ visiting; prison visitation systems being not very family friendly is a point that has commonly been brought up in the literature (e.g., Christian, 2005; Nesmith and Ruhland, 2008; Chui, 2010; Galardi et al., 2015), and one that should be worth addressing. However, it should be acknowledged that a considerable number of participants who reported a rather strained and dysfunctional family relationship with their partner and children prior to their incarceration did not seem to voice this concern in the present study.

The next two aspects of ambiguous loss and boundary ambiguity seemed to be somewhat related in the current context. It was firstly found that ambiguous loss was a common issue for families of the incarcerated, as the majority of participants either chose to withhold information regarding their incarceration from their children, or chose not to approach the topic directly and as such remained unsure of how much their children actually knew. As above, the finding section illustrates that some participants were unwilling or reluctant to inform children about their involvement in crime and history of imprisonment. Some attributed it to the cultural stigma surrounding prison and others worried about the negative consequences associated with such a disclosure, such as losing respect from the young ones. In any case, the question of whether or not children should be exposed to the truth of their parent’s incarceration – and how exactly to appropriately do so – is a debate that persists in the literature, with some even referring to the disclosure of information as a “conspiracy of silence” (e.g., Kampfner, 1995; Parke and Clarke-Stewart, 2001). The ambiguous loss observed in this study partly also tied in with boundary ambiguity, as the ambiguity surrounding the father’s incarceration served as an additional barrier – on top of the actual imprisonment – between father and children. This notwithstanding, participants commonly reported distancing themselves from family members, blurring their role as a father in the family, which resulted in parenting-related problems both during and after their incarceration. The narratives in the form of direct quotations, in the finding section above, show that while most of them wanted to continue to perform their fathering role, almost all experienced frustration and a sense of helplessness in their attempt to show their concern and care to family members. They, however, understood very well that the physical barrier affected the emotional ties with other family members during incarceration, and some were particularly stressed about how their family relationships could be restored after imprisonment. These findings echo those from previous works, providing strength to the argument that rehabilitation strategies must, along with the other mentioned recommendations, also prioritize helping participants retain and strengthen their fatherhood identity throughout the entire process for the sake of family cohesion (Sampson and Laub, 1997; Hairston, 2001; Uggen et al., 2005; Dyer et al., 2012). Moreover, the participants in this study that were able to successfully hold on to their sense of fatherhood did so by being honest and forthcoming with their children, even leveraging their experiences as life lessons, implying that boundary ambiguity cannot be addressed without first dealing with ambiguous loss.

This study is not without limitations. Data was collected solely from the incarcerated fathers and not their family members, and as such may have been biased from their perspective. Moreover, several incarceration-specific factors such as the type of offense, length of the sentence and number of repeat offenses were not taken into account in the analysis of the data. This was due to the somewhat homogenous nature of the sample recruited, in that it consisted mainly of drug offenders, as well as the relatively modest sample size. As such, the findings here may not be generalizable to the entire prison population – it is plausible that more severe offenses such as murder might lead to worse family detachment due to the heinous nature of the crime. The inclusion of such factors in further research would no doubt be helpful in uncovering the exact circumstances under which the family process holds weight, or how elements of the theory might vary under different contexts. Nonetheless, the findings of the current study should at least be relevant to families of the incarcerated who remain connected, or at least hold some desire to be.

In summary, more needs to be done to provide support for families during the transition phase following the incarceration to facilitate their rules of transformation, assist their establishing of family rituals by improving the visitation system, and encourage the minimization of ambiguous loss and boundary ambiguity to help inmates retain their fatherhood identity and promote family cohesion. Altogether, these recommendations aim to help families attain some sense of normalcy and reach a state of equilibrium, proposed by family process theory to be integral to the long-term functioning of a family (Broderick, 1993).

All in all, this study provides support for the application of family process theory to understand the challenges faced by the families of the incarcerated, offers some qualitative evidence from a small number of male ex-prisoners within the Chinese context, and puts forth a strong justification for further research and practice to adopt a more family centric approach. This should be done to not only alleviate the burdens associated with incarceration, but even possibly help affected individuals grow stronger from it together. It is important to recognize that families need not be viewed merely as something to be protected in the event of an incarceration, but rather that families can even serve as an asset to inmates’ personal development and rehabilitation given the right circumstances. Indeed, the current study found that the fear of being separated again from one’s family helped inmates develop more selfless tendencies and acted as an effective deterrent to their further involvement in crime. Moreover, as implied throughout the entirety of this study and touched on in the literature, love and support are key components in helping released inmates get back on their feet, which families can be an integral source of Uggen et al. (2005). This may particularly be the case in Chinese contexts given the value placed on kinship and family ties. It is likely that participants recognized this, which was perhaps why many of them expressed that more focus should be placed on assisting inmates families’ as a whole to preserve their integrity, a sentiment that the author wholeheartedly agrees with.

## Author Contributions

The author confirms being the sole contributor of this work and approved it for publication. He would like to thank Mr Andrew Yeung and Miss Aiden Tong for their support and research assistance with this project.

## Conflict of Interest Statement

The author declares that the research was conducted in the absence of any commercial or financial relationships that could be construed as a potential conflict of interest. The reviewer EH and handling Editor declared their shared affiliation, and the handling Editor states that the process nevertheless met the standards of a fair and objective review.
